# Epilepsy Surgery: Factors That Affect Patient Decision-Making in Choosing or Deferring a Procedure

**DOI:** 10.1155/2013/309284

**Published:** 2013-09-16

**Authors:** Christopher Todd Anderson, Eva Noble, Ram Mani, Kathy Lawler, John R. Pollard

**Affiliations:** ^1^Department of Neurology, Medical College of Wisconsin, 9200 West Wisconsin Avenue, FWC, Milwaukee, WI 53226, USA; ^2^Johns Hopkins School Public Health, JHU, Baltimore, MD 21224, USA; ^3^Department of Neurology, University of Medicine and Dentistry of New Jersey, New Brunswick, NY 08901, USA; ^4^Department of Neurology, Penn Epilepsy Center, University of Pennsylvania, HUP, Philadelphia, PA 19104, USA

## Abstract

Surgical resection for well-selected patients with refractory epilepsy provides seizure freedom approximately two-thirds of the time. Despite this, many good candidates for surgery, after a presurgical workup, ultimately do not consent to a procedure. The reasons why patients decline potentially effective surgery are not completely understood. We explored the socio cultural, medical, personal, and psychological differences between candidates who chose (*n* = 23) and those who declined surgical intervention (*n* = 9). We created a novel questionnaire addressing a range of possible factors important in patient decision making. We found that patients who declined surgery were less bothered by their epilepsy (despite comparable severity), more anxious about surgery, and less likely to listen to their doctors (and others) and had more comorbid psychiatric disease. Patients who chose surgery were more embarrassed by their seizures, more interested in being “seizure-free”, and less anxious about specific aspects of surgery. Patient attitudes, beliefs, and anxiety serve as barriers to ideal care. These results can provide opportunities for education, treatment, and intervention. Additionally, patients who fit a profile of someone who is likely to defer surgery may not be appropriate for risky and expensive presurgical testing.

## 1. Introduction

 Approximately one-third of patients with epilepsy will not attain seizure freedom through medications alone. If patients are not free of seizures after trying two different antiepileptic drugs (AEDs), their chance of relief from a third is between five and ten percent [1].

After multiple AEDs have failed, physicians often propose resective epilepsy surgery. In 2001, a randomized, controlled trial comparing temporal lobectomy to continued pharmacologic therapy found that 58% of the surgical group was seizure-free at one year, compared with 8% in the pharmacologic group [2]. These results have been replicated, and meta-analyses show that approximately two-thirds of patients are seizure-free at long-term followup after a temporal lobe resection [3, 4]. 

There are more candidates for epilepsy surgery than those who actually receive a procedure. There are many factors involved in this disparity: patients' lack of access to comprehensive epilepsy surgery centers, low levels of referrals by physicians, and an unclear medical consensus regarding the appropriateness of recommending surgery versus continued AED treatment [5]. Even when referred to an epilepsy center, some good candidates for epilepsy surgery decline the procedure and choose to remain on pharmacologic therapy, even after undergoing a tedious and expensive presurgical evaluation. Patients' attitudes towards surgical intervention likely affect procedure rates although the literature on this barrier is limited.

 Swarztrauber et al. [6] held focus groups to ask patients questions about their views of different treatments for intractable epilepsy, including surgery. The authors found many patients opted against surgery due to a general mistrust of brain surgery, negative medical provider opinions of surgery, lack of correct information about the success rates and risks, and poor patient-physician relationships. We conducted this study to further explore how sociocultural, medical, personal, and neuropsychological factors are associated with patients' decision making regarding surgery.

 We studied the attitudes, priorities, and beliefs of patients who were offered a temporal or extratemporal resection and compared the responses of those who chose the surgery (surgical group) to those who decided against it (nonsurgical group). We used a survey to collect patients' opinions and carried out a chart review of relevant medical information. Our goal was to determine what issues should be addressed with these patients throughout the process of presurgical evaluation to prevent unnecessary testing and help good candidates make appropriate, evidence-based decisions.

## 2. Materials and Methods

### 2.1. Participants

We recruited patients from the Penn Epilepsy Center at the Hospital of the University of Pennsylvania over a 12-month interval. Inclusion criteria were as follows: all patients had to be 18 years or older, have intractable partial-onset epilepsy, had undergone a presurgical workup including a stay in the epilepsy monitoring unit, an MRI of the brain, an FDG-PET of the brain, neuropsychological testing specific to epilepsy, and were offered either resective epilepsy or invasive electrocorticography with a plan of likely resection at explant of intracranial electrodes. All patients provided informed consent. All study methods were approved by the hospital's IRB and in accordance with HIPAA regulations.

While we aimed for an equal number of participants in the surgical and nonsurgical groups, there were challenges recruiting in the nonsurgical group, such as being unable to contact patients or follow up initial conversations by phone (detailed further in the discussion). We initially contacted 41 patients in total, 26 surgical patients and 15 nonsurgical patients. Thirty-three participated, for a participation rate of 80.5%. When we attempted to follow up our initial conversation with eight patients, we were unable to contact them further. Twenty-three of the respondents were in the surgical group, and ten were in the nonsurgical group (One patient who refused surgery had to be retrospectively excluded from the data analysis due to a lack of some pre-surgical tests, so all analysis was done with *n* = 9).

Patients were invited to participate either by phone or directly after their regular clinic appointment. Whether conducted via telephone or in person, the study survey lasted approximately 30 minutes (range: 15–60 minutes). Twenty-one surveys were completed in person (100% response rate), and 12 surveys were completed over the phone (60% response rate). 

### 2.2. The Questionnaire

Due to the lack of survey instruments designed for the purpose of assessing epilepsy patients' attitudes towards surgery, we created a novel questionnaire to gain this subjective information. (See Appendix for the questionnaire). 

 We also collected demographic information: age, level of education, place of birth, marital status, self-reported ethnicity, number of children, employment status, disability status, and other surgical histories. We asked about epilepsy characteristics: age at seizure onset, duration of epilepsy, number of current AEDs, number of previous AEDs, and seizure frequency. 

Patients were presented with a list of 36 potentially important factors to their decision-making process and rated each on a Likert scale from 0 (not important) to 4 (most important) or “Not Applicable.” Each factor fell under one of the following themes:

- Details of Epilepsy,
- Effects of Epilepsy,
- Other People/Group's Beliefs,
- Surgical Fears,
- Medication Effects,
- Hopes After Surgery,
- Alternative Treatment Options,
- Personal Beliefs About the Surgery,
- Doctor's Information About Surgery.

For “Details of Epilepsy,” we asked patients to consider how long they had been living with epilepsy and the frequency and severity of their seizures. “Effects of Epilepsy” factors included work limitations from seizures, stigma of having epilepsy, embarrassment from seizures in public, fear of death from seizures, fear of physical injury from seizures, the desire/need to be seizure-free, and access to disability benefits. “Other People/Group's Beliefs” included the opinions of family members and friends and the effect of faith and religion. “Surgical Fears” included fear of surgery in general, comfort of surgery in general, fear of being put under anesthesia, fear of not waking up after the procedure, fear of complications during the procedure, fear of resulting memory loss and/or cognitive decline, and concerns that other health conditions may impact the surgery. “Medication Effects” included the number of medications taken prior to surgery (or proposed surgery) and the physical and mental side effects of antiepileptic medications. “Hopes After Surgery” addressed career opportunities and the future ability to drive. “Alternative Treatment Options” included availability of the vagal nerve stimulator and future devices such as deep brain stimulation or responsive brain stimulation, which may reach the marketplace in the next several years [7]. “Personal Beliefs About the Surgery” factors were the personal belief that the surgery would work, knowledge of others' successes or failures with a procedure, and the extent to which surgery has been proven scientifically. “Doctor's Information About Surgery” factors were the chances of success, risks of complications during surgery, and the risks of disability after surgery; all quoted to the patient by the physician or neurosurgeon. Finally, patients were invited to list any factors that influenced their decision but were not covered in the survey. 

Last, we asked participants where they got most information about the surgical procedure and who was most influential in their decision. The available choices were epilepsy doctor, neurosurgeon, another member of the epilepsy center team, informational DVD, another patient who had the procedure, family or friends, or other.

### 2.3. Chart Review

We conducted a chart review to objectively gather the following information: epilepsy history and diagnosis, drug and alcohol history, cause of epilepsy, type of seizures, and comorbid psychiatric disorders.

### 2.4. Statistical Analysis

Ordinal data was compared using the Wilcoxen rank-sum test. Categorical data was compared using Fisher's test. *P* values of less than 0.05 were considered indicative of significant group differences.

## 3. Results

### 3.1. Demographics

Tables 1 and 2 report demographic characteristics across our surgical and nonsurgical groups. There was a significant difference in age (mean surgical age = 43, mean nonsurgical age = 54, *P* = 0.046), self-reported ethnicity (*P* = 0.015), and comorbid psychiatric disorders (*P* = 0.005). Several other variables did not reach statistical significance.

### 3.2. Factors Affecting the Surgical Decision

For our analysis, we grouped together responses of “Not Applicable” and “Not Important.” We assigned both responses to our 36 factors a score of 0 in our calculations.  Table 3 reports the means and standard deviations for the individual factors and themes.

Twelve factors revealed significant differences between the two groups: frequency and severity of seizures (*P* = 0.002), length of time with seizures (*P* = 0.031), stigma of having epilepsy (*P* = 0.022), embarrassment from seizures in public (*P* = 0.037), need/desire to be seizure-free (*P* = 0.000), frustration with epilepsy (*P* = 0.000), general comfort with surgery (*P* = 0.019), fear of surgery in general (*P* = 0.005), fear of complications during surgery (*P* = 0.002), concerns that my other health conditions may impact surgery (*P* = 0.045), chances of success quoted to me by my doctor (*P* = 0.040), and my belief that surgery would work (*P* = 0.002). 

### 3.3. Patient Sources of Information and Influences

Table 4 reports patients' sources of information about surgery, and identifies the most influential individual in patients' decision-making process. Options that were not chosen by any patients were not included in the table and frequently given answers by patients who chose “Other” were included. Patients were allowed to choose multiple answers for each question.

The majority of patients identified their epilepsy doctor as the main source of information (*n* = 28). Other common sources were the neurosurgeon (*n* = 8) and the Internet (*n* = 6). The majority of patients also said their epilepsy doctor was the most influential individual in their decision (*n* = 18). Other important influences were the neurosurgeon (*n* = 8), themselves (*n* = 8), family and friends (*n* = 7), and a former patient (*n* = 1). 

Though not significant, there were interesting trends in the patients' responses. Surgical patients were more likely than nonsurgical patients to report that they gained most of their information from the Internet (27.1% versus 11.1%), neurosurgeon (34.8% versus 0%), or epilepsy doctor (52.2% versus 33.3%). Nonsurgical patients were more likely than surgical patients to select “myself” as the most influential individual (55.6% versus 13%).

## 4. Discussion 

This study was an exploratory/hypothesis generating study to determine why some candidates for epilepsy surgery refuse surgery. We had a small sample size and created a new questionnaire specifically for this study; these results should be viewed as first step; further research should be done to explore the reasons behind these differences. We surmise that refusal of recommended epilepsy surgery is a more widespread problem than most clinicians are aware. 

The first finding from our study was that patients who completed a presurgical evaluation but chose against surgery are often difficult to contact and/or unwilling to discuss their decisions. We aimed for approximately 25 participants in each group but were unable to complete the interview with more than 9 nonsurgical patients. We found they often had nonworking phone numbers listed with the hospital, they were not responsive to voicemails or letters, and if we successfully made contact and they agreed to participate, they would miss phone and clinic appointments. As a result, we were only able to talk to patients who had recently made a decision, usually at their clinic appointments. 

Carlson et al. also noticed that in their cohort of surgical candidates many did not progress to surgery [8]. With some findings comparable to ours they found that 21% of their surgical candidates declined surgery, 16% had no identifiable reason, and 25% were lost to followup. 

### 4.1. Demographics

Looking at our study population, the surgical and nonsurgical groups differed in age, AED exposure, ethnicity, and comorbid psychiatric disorders. We also found a difference in self-reported ethnicity between groups—only 8.7% of the surgical group were African American, compared with 44.4% of the nonsurgical group. Swarztrauber et al. (2003) found that African American patients were less likely to opt for surgery, and while our study does not indicate causation, this difference was significant. Lastly, nonsurgical patients had a much higher prevalence of psychiatric disorders, excluding depression (which was equally prevalent between groups). Almost half the nonsurgical group had severe anxiety, while only 4.4% of the surgical group did. Perhaps this presence of anxiety was affecting patients' decisions. Interestingly, seizure types and frequency were not different between groups, indicating that the nonsurgical group was not strongly influenced by relatively less frequent or disruptive seizures. Although doses of antiepileptic drugs were not tracked, the number of agents and choice of drugs were comparable between the two groups. At the time of their decision to choose or defer surgery, all patients were on between 1 and 3 antiepileptic drugs. As seen in Table 2 there was no significant difference in the number of drugs that patients were on at the time of their decision; thus it is unlikely that cognitive side effect had a major impact on the patients' choices. The most commonly administered agents were levetiracetam, lamotrigine, and carbamazepine. None of these are associated with severe cognitive slowing often seen with barbiturates or benzodiazepines. None of our patients were on either of those two classes of AED.

### 4.2. Decision-Making Factors

Of our 36 factors, 12 differed significantly. Frequency and severity and length of time with seizures were more important factors in the decision for the surgical group. Furthermore, the surgical group cited the need/desire to be seizure-free, the stigma of epilepsy, embarrassment from seizures in public, and frustration with epilepsy as more important than the nonsurgical group, indicating perhaps greater sensitivity in the surgical group to the adverse social effects of epilepsy. 

The nonsurgical group reported that surgical fears were more influential factors in their decision. Specifically, patients in the nonsurgical group rated a general comfort (or discomfort) with surgery, fear of surgery in general, fear of complications during surgery, and concerns about complications due to comorbidities as significantly more important than did the surgical group. Often, patients elaborated their reasons here, expressing that the surgery was too risky to have without an absolute guarantee of seizure freedom. This is an important difference between groups because if a patient expresses intense fear of surgery, this could be addressed with multiple conversations with various providers, support groups, and other patients. Instead of suggesting surgery once, as is often the case, and assuming patients will understand that surgery offers the greatest chance of seizure freedom and make a decision based on our statistical logic, physicians might more gradually introduce the idea of surgery. Also, if a patient continuously expresses these surgical fears, the physician should recognize and respect the pervasive fear of surgery in patients and prevent unnecessary tests often done in a complete presurgical evaluation. Although we did not track this variable objectively, it was clear through chart review and by familiarity with the cohort that the offering and recommendation to go to surgery were typically attempted multiple times and were without change in the patient's attitude. This raises the question of whether more discussions or exposure to epilepsy surgery would actually help the patients get a recommended procedure.

 Other significant differences across groups were the chances of success quoted by doctors and a personal belief that surgery would work. The surgical group found the quoted success rate more important, while the nonsurgical group believed that the quoted success rate was too low for such an invasive procedure. The surgical group also said their belief the surgery would work was very important to them, while the nonsurgical group, predictably, did not.

 Overall, our results indicate that our nonsurgical patients are less sensitive to the effects of their epilepsy, have greater fears of surgery, find statistical success rates less important, and do not believe surgery would work for them. These beliefs start to create a patient profile that physicians could use in the future to assess whether a patient will or will not choose surgery. This profile, though it needs elucidation with additional larger studies, could affect the physician's decision to suggest presurgical testing or not. 

### 4.3. Patient Sources of Information and Influences

Questions regarding sources of information and influences on decisions showed a trend. The nonsurgical group was less likely to identify their doctor as most influential in their decision, and many more identified themselves as most influential. This could mean that there is an opportunity for epilepsy doctors to be more important in the decision-making process by increasing the number of conversations, following up more with patients, and otherwise building a strong rapport with patients so they will trust and follow medical recommendations. Such is of course conjecture; the nonsurgical patients may have a more intransigent style, and more exposure or dialogue may either have no benefit or may make them more entrenched in their beliefs which are discordant with those of their clinicians. 

## 5. Conclusions

Access to medical care is a major issue in all branches of medicine and in epilepsy as well [9]. Typically researchers focus on lack availability of care or poor referral patterns [10]. We focused on patient attitudes as a barrier to care which we believe is a neglected variable. Our study reveals differences between patients who choose to have surgery and those who defer. These differences are social, personal, medical, and psychiatric and help create a profile of patients who are more likely opt against a procedure despite medical need and physician recommendation. Although our study had small statistical power due to the small sample sizes these findings may be helpful in generating further hypotheses on why patients decline recommended care, assisting in understanding the process of patient choice and, through open discussion, prevent unnecessary presurgical evaluations for patients who are adamantly against surgery regardless of medical opinion.

## Figures and Tables

**Table 1:** Demographic characteristics of participants.

	Surgical group (*n* = 23)	Nonsurgical group (*n* = 9)	*P* value
Age (years)			
Median (IQR)	43 (32–47)	54 (42–56)	**0.046**
Education (years)*			
Median (IQR)	14 (12–16)	12 (12–14)	0.163
Birthplace			1.000
NJ	2 (8.7%)	0 (0%)	
PA	18 (78.3%)	8 (88.9%)	
Other in the USA	2 (8.7%)	1 (11.1%)	
Outside the USA	1 (4.3%)	0 (0%)	
Current location			1.000
NJ	2 (8.7%)	1 (11.1%)	
PA	21 (91.3%)	8 (88.9%)	
Marital status			1.000
Single	9 (39.1%)	4 (44.4%)	
Married	12 (52.2%)	5 (55.6%)	
Divorced	2 (8.70%)	0 (0%)	
Children			1.000
No	11 (47.8%)	4 (44.4%)	
Yes	12 (52.2%)	5 (55.6%)	
Self-reported ethnicity			**0.016**
White	20 (87.0%)	4 (44.4%)	
Black	2 (8.7%)	4 (44.4%)	
Indian	1 (4.3%)	0 (0%)	
Hispanic	0 (0%)	1 (11.1%)	
Currently employed			0.243
No	9 (39.1%)	6 (66.7%)	
Yes	14 (60.9%)	3 (33.3%)	
On social security disability benefits			0.109
No	16 (69.6%)	3 (33.3%)	
Yes	7 (30.4%)	6 (66.7%)	
Comorbid depression			0.694
No	11 (47.8%)	3 (33.3%)	
Yes	12 (52.2%)	6 (66.7%)	
Other comorbid psychiatric disorders			**0.005**
None	19 (82.6%)	4 (44.4%)	
Anxiety	1 (4.4%)	4 (44.4%)	
Mood disorder	1 (4.4%)	1 (11.1%)	
ADHD	2 (8.7%)	0 (0%)	
History of alcohol or illegal drug use			1.000
No	22 (95.6%)	9 (100%)	
Yes	1 (4.4%)	0 (0%)	
History of smoking cigarettes			1.000
No	19 (82.6%)	7 (77.8%)	
Yes	4 (17.4%)	2 (22.2%)	
Prior surgical history			0.249
No	11 (47.8%)	2 (22.2%)	
Yes	12 (52.2%)	7 (77.8%)	
Prior bad surgical experience			1.000
No	20 (87.0%)	8 (88.9%)	
Yes	3 (13.0%)	1 (11.1%)	

Ordinal data was compared using the Wilcoxen rank-sum test. Categorical data was compared using Fischer's test. *P* values <0.05 considered significant. *12 years education = high school graduate.

**Table 2:** Epilepsy characteristics of participants.

	Surgical group (*n* = 23)	Nonsurgical group (*n* = 9)	*P* value
Total years with seizures (years)			
Median (IQR)	11 (9–21)	14 (10–35)	0.487
Age at onset of seizures (years)			
Median (IQR)	22 (13–26)	21 (14–44)	0.425
Years since procedure or decision			
Median (IQR)	4 (2–7)	1 (1-1)	**0.000**
Current number of AEDs being taken			
Median (IQR)	1 (1-2)	2 (2-3)	0.082
Total AEDs tried until procedure or decision			
Median (IQR)	5 (3–6)	6 (5–7)	0.056
Seizure frequency (per month)			0.287
1–3	6 (26.1%)	4 (44.4%)	
4–9	5 (21.7%)	2 (22.2%)	
10+	12 (52.2%)	3 (33.3%)	
Types of seizures			0.327
Complex partial	12 (52.2%)	2 (22.2%)	
Generalized tonic clonic (GTC)	0 (0%)	0 (0%)	
Simple partial and complex partial	2 (8.7%)	2 (22.%)	
Simple partial and GTC	1 (4.3%)	1 (11.1%)	
Complex partial and GTC	6 (26.1%)	2 (22.2%)	
Simple partial, complex partial, and GTC	2 (8.7%)	2 (22.2%)	
Proposed/completed procedure			0.308
Left temporal lobectomy	10 (43.5%)	6 (66.7%)	
Another left resection	1 (4.3%)	0 (0%)	
Right temporal lobectomy	11 (47.8%)	2 (22.2%)	
Another right resection	1 (4.3%)	0 (0%)	
Other	0 (0%)	1 (11.1%)	
Cause of epilepsy			0.694
Cryptogenic (including mesial temporal sclerosis)	16 (69.6%)	6 (66.7%)	
Traumatic brain injury	5 (21.7%)	1 (11.1%)	
Congenital brain abnormality	2 (8.7%)	2 (22.2%)	

Ordinal data was compared using the Wilcoxen rank-sum test. Categorical data was compared using Fischer's test. *P* values <0.05 considered significant.

**Table 3:** Importance of factors in epilepsy surgery decision making.

	Surgical group (*N* = 23)	Nonsurgical group (*N* = 9)	*P* value
Details of epilepsy			
Frequency and severity of seizures	3.48 (0.8)	1.89 (1.4)	**0.002**
How long I have had seizures	2.957 (1.4)	1.56 (1.7)	**0.031**
Effects of epilepsy			
Work limitations as a result of seizures	2.435 (1.7)	1.44 (1.4)	0.082
The stigma of having epilepsy	2.435 (1.8)	0.89 (1.8)	**0.022**
Embarrassment from seizures in public	2.522 (1.6)	1.11 (1.7)	**0.037**
Fear of death from seizures	1.652 (1.5)	0.89 (1.4)	0.154
Fear of physical injury from seizures	2.435 (1.6)	1.44 (1.2)	0.081
Need/desire to be seizure-free	3.783 (0.6)	2.0 (1.2)	**0.000**
Frustration with epilepsy	3.522 (0.6)	1.33 (1.4)	**0.000**
Disability benefits	0.869 (1.4)	0.56 (1.0)	0.747
Other people or group's beliefs			
Opinions of family members	2.0 (1.6)	2.44 (1.7)	0.436
Opinions of friends	1.261 (1.4)	0.67 (1.0)	0.355
My faith or religion	1.565 (1.8)	0.78 (1.6)	0.236
Surgical fears			
General comfort with surgery	2.0 (1.5)	3.33 (0.9)	**0.019**
Fear of surgery in general	1.391 (1.4)	3.11 (1.2)	**0.005**
Fear of being put under anesthesia	0.696 (1.3)	1.22 (1.5)	0.218
Fear that I will not wake up after surgery	1.086 (1.5)	1.67 (1.7)	0.254
Fear of complications during surgery	1.739 (1.5)	3.56 (0.5)	**0.002**
Fear of memory (or other) cognitive problems after surgery	2.478 (1.5)	3.0 (1.7)	0.172
Concerns that my other health conditions may impact surgery	0.652 (1.2)	2.11 (2.0)	**0.045**
Hopes after surgery			
Career opportunities	2.13 (2.3)	1.56 (1.7)	0.399
Future ability to drive	2.652 (1.7)	1.78 (1.8)	0.143
Doctor's information about surgery			
The chances of success quoted to me by my doctor	3.304 (1.1)	2.33 (1.4)	**0.040**
The risk of complications during surgery quoted to me by my doctor	2.13 (1.4)	2.56 (1.5)	0.413
The risk of disability after surgery quoted to me by my doctor	1.565 (1.4)	2.22 (1.9)	0.289
Personal beliefs about the procedure			
My own understanding of the surgical procedure	2.565 (1.1)	2.78 (1.2)	0.602
My belief that surgery would work	3.565 (0.7)	2.22 (1.2)	**0.002**
Knowledge of others' successes or failures with epilepsy surgery	1.348 (1.6)	1.78 (1.6)	0.448
The degree to which surgical treatment is proven scientifically	2.739 (1.1)	2.67 (1.3)	0.982
Medication effects			
The number of medications I take (or took presurgery)	2.869 (1.4)	2.22 (1.3)	0.150
Physical side effects of seizure medications	2.174 (1.4)	1.78 (1.6)	0.467
Cognitive or emotional side effects of seizure medication	2.13 (1.6)	1.56 (1.7)	0.354
Other treatment options			
Alternative treatments that may be available to me in the near future	0.522 (1.1)	1.44 (1.8)	0.184
Availability of the vagal nerve stimulator	0.348 (0.9)	0.78 (1.2)	0.229
The surgeon recommended the vagal nerve stimulator	0.348 (0.8)	0.44 (1.3)	0.765
The vagal nerve stimulator seemed safer	0.251 (0.8)	0.67 (1.3)	0.445

Values reported as mean (standard deviation). *P* value provided by the Wilcoxen rank-sum test.

**Table 4:** Patient sources of information and influences.

	Surgical group (*N* = 23)	Nonsurgical group (*N* = 9)	*P* value
Where did the patient gain the majority of their information regarding the surgical procedure?			0.667
My epilepsy doctor	20 (87.0%)	8 (88.9%)	
My neurosurgeon	7 (30.4%)	1 (11.1%)	
A former patient who had surgery	1 (4.3%)	0 (0%)	
Viewing the informational DVD, provided by the Penn Epilepsy Center	1 (4.3%)	0 (0%)	
My family and friends	1 (4.3%)	0 (0%)	
The internet	5 (21.7%)	1 (11.1%)	
Who is most influential in the patient's decision?			0.234
My epilepsy doctor	12 (52.2%)	3 (33.3%)	
My neurosurgeon	8 (34.8%)	0 (0%)	
A former patient who had surgery	1 (4.3%)	0 (0%)	
My family and friends	4 (17.4%)	3 (33.3%)	
Myself	3 (13.0%)	5 (55.6%)	

Patients were allowed to choose multiple options; therefore, the column totals do not equal the total patients in each group. *P* value calculated with Fischer's exact test.

## Appendix

### A. Decision Making in Epilepsy Questionnaire

The following questionnaire is designed to assess why people chose to have epilepsy surgery or why they chose to defer such an operation. We thank you for your time and participation.

#### A.1. Demographic Information:

 Patient number: —————

Place of Birth: ——————

State of residence: —————

Level of education: —————

Marital Status: —————

Number of children (if any): —————

My ethnicity is best described as: —————

Did you require help to complete this form? (Circle your choice)

YES
NO

#### A.2. Questions about Your Seizures:

Please circle the best answer:

 (1) My seizures are dangerous

TRUE
FALSE

 (2) My seizures are disabling

TRUE
FALSE

 (3) My seizures disrupt my life but are not severe

TRUE
FALSE

 (4) My seizures bother me only slightly

TRUE
FALSE

 (5) My seizures do not bother me at all

TRUE
FALSE

I have had seizures for how many years: ———————

Age of onset of seizures: ——————

Frequency of seizures: ——————

(per week or per month or per year)

Are you currently employed: (circle your choice)

YES
NO

Are you currently on disability? (circle your choice)

YES
NO

Current medications: ————————————————————————————————————————————————————————————————————————————————————————————————————————

Did you choose to have epilepsy surgery?

(circle your choice)

YES
NO

Please rank how much each of the following influenced your decision about surgery. Please choose a response from 0 to 4 for each item. 0 means it was not important to you in your decision, and 4 means it was very important to you. If the factor does not relate to you, please choose N/A for Not Applicable. If you chose to have surgery, these questions are regarding these factors **before surgery**.

#### A.3. Factors in Choosing to Have or Not Have Surgery:

not important → most important

Frequency and severity of seizures:

N/A
0
1
2
3
4

Work limitations as a result of seizures:

N/A
0
1
2
3
4

How long I've had seizures:

N/A
0
1
2
3
4

The stigma of having epilepsy:

N/A
0
1
2
3
4

Embarrassment from seizures in public:

N/A
0
1
2
3
4

Fear of death from seizures:

N/A
0
1
2
3
4

Fear of physical injury from seizures:

N/A
0
1
2
3
4

Need/desire to be seizure-free:

N/A
0
1
2
3
4

Frustration with epilepsy:

N/A
0
1
2
3
4

Career opportunities:

N/A
0
1
2
3
4

Disability benefits:

N/A
0
1
2
3
4

My belief that surgery would work:

N/A
0
1
2
3
4

Opinions of family members:

N/A
0
1
2
3
4

Opinions of friends:

N/A
0
1
2
3
4

My faith or religion:

N/A
0
1
2
3
4

Knowledge of other's successes or failures with epilepsy surgery:

N/A
0
1
2
3
4

The chances of success quoted to me by my doctor:

N/A
0
1
2
3
4

The risk of complications during surgery quoted to me by my doctor:

N/A
0
1
2
3
4

The risk of disability after surgery quoted to me by my doctor:

N/A
0
1
2
3
4

General comfort with surgery:

N/A
0
1
2
3
4

Fear of surgery in general:

N/A
0
1
2
3
4

Fear of being put under anesthesia:

N/A
0
1
2
3
4

Fear that I won't wake up after surgery:

N/A
0
1
2
3
4

Fear of complications during surgery:

N/A
0
1
2
3
4

Fear of memory (or other) cognitive problems after surgery:

N/A
0
1
2
3
4

Concerns that my other health conditions may impact surgery:

N/A
0
1
2
3
4

Future ability to drive:

N/A
0
1
2
3
4

My own understanding of the surgical procedure:

N/A
0
1
2
3
4

The number of medications I take (or took pre-surgery):

N/A
0
1
2
3
4

Physical side effects of seizure medications:

N/A
0
1
2
3
4

Cognitive or emotional side effects of seizure medication:

N/A
0
1
2
3
4

Alternative treatments that may be available to me in the near future:

N/A
0
1
2
3
4

The degree to which surgical treatment is proven scientifically:

N/A
0
1
2
3
4

Availability of the vagal nerve stimulator:

N/A
0
1
2
3
4

The surgeon recommended the vagal nerve stimulator:

N/A
0
1
2
3
4

The vagal nerve stimulator seemed safer:

N/A
0
1
2
3
4

Other reasons: ————————

N/A
0
1
2
3
4

#### A.4. Additional Questions Regarding Surgical Choice:

  (1) Where did you gain most of your information regarding surgery:

- My epilepsy doctor
- My neurosurgeon
- Another member of the epilepsy center team
- A former patient who chose to have surgery
- Viewing the DVD (get name here?)
- My family and friends
- Other: —————

 (2) Who was most influential in your decision to have, or not have, surgery?

- My epilepsy doctor
- My neurosurgeon
- Another member of the epilepsy center team
- A former patient who chose to have surgery
- Viewing the DVD (get name here?)
- My family or friends
- Other: —————

 (3) How many different hospitals have discussed epilepsy surgery with you?

(Please circle one)
1
2
3
More than 3

 (4) Do you feel that all of your questions regarding the proposed surgical procedure were answered?

YES
NO

 (5) Have you had surgery before?

YES
NO

 (6) Have you had a bad experience with surgery before?

YES
NO

If yes, please explain: ——————

#### A.5. Items for Completion by Research Team:

*Epilepsy Surgery*

Procedure that was done: (to be filled in by MD)

———————————

Procedure that was not done: (to be filled in by MD)

—————————

Number of medication trials: —————

Etiology of epilepsy: ———————

Types of seizures: ———————

Comorbid depression: ——————

YES
NO

Other psychiatric disease: ——————

YES
NO

Does the doctor perceive the patient as mistrustful? ——

YES
NO

Is the patient well-adherent with medical plans? ————

YES
NO

Neuropsychological testing results: ——————

IQ: ——————

Does the patient use illegal drugs: ——————

Does the patient smoke: ——————

Does the patient drink alcohol excessively: ——————

Wada results: ————————————

MRI results: ————————————

PET results: ————————————
